# 9 years’ follow-up of 168 pin-fixed supracondylar humerus fractures in children

**DOI:** 10.1080/17453674.2018.1438765

**Published:** 2018-02-16

**Authors:** Noora Tuomilehto, Antti Sommarhem, Aarno Y Nietosvaara

**Affiliations:** 1 Department of Orthopedics and Traumatology, Helsinki University; 2 Children’s Hospital, Helsinki University Central Hospital, Helsinki, Finland

## Abstract

**Background and purpose:**

The long-term outcome of pin-fixed supracondylar humerus fractures (SCHF) in children is not well known. We assessed the 7- to 12-year outcome in 168 children.

**Patients and methods:**

During 2002–2006, 210 domestic children (age 7 (1–14) years) with SCHF (Gartland III 79%, Gartland II 19%, and flexion type 2%) were pin fixed in Helsinki. 36 (17%) patients had a nerve palsy. Radiographic alignment was regarded as satisfactory in 81% of patients (Baumann angle (BA) within ±10˚ of normal range and whose anterior humeral line (AHL) crossed the capitulum). After a mean follow-up of 9 (7–12) years, 168 (80%) patients answered a questionnaire regarding elbow appearance (scale 0–10), function (scale 0–10), and pain (scale 0–10), and symmetry of range of motion (ROM) and carrying angle (CA). 65 (31%) patients also attended a clinical follow-up examination.

**Results:**

Mean subjective score for appearance was 8.7 (2–10) and for function 9.0 (2–10) (n = 168). Elbow ROM asymmetry was experienced by 28% and elbow CA asymmetry by 17% of the patients. Elbow pain was reported by 14%, and was more common in children with nerve injuries. Long-term outcome was good or excellent in 60/65 and CA in 56/65 of the follow-up visit patients using Flynn’s criteria. BA exceeding normal values by 10˚ was associated with lower subjective outcome; AHL crossing point with the capitulum was not associated with outcome.

**Interpretation:**

Long-term subjective outcome is satisfactory with few exceptions if elbow ROM and CA are restored within 10° of the uninjured elbow. Radiographs at fracture union have little prognostic value. Nerve injuries can cause long-term pain.

Displaced supracondylar humerus fractures in children (SCHF) have a high risk of fracture or treatment-related complications. Displaced SCHF is best treated with internal fixation and is thus the most common operatively treated fracture type in children (Cheng et al. 1999, Omid et al. 2008). Quality of SCHF treatment correlates with the experience of the treating institution: unsatisfactory standard of reduction and pin fixation are the most common reasons for treatment-related nerve injuries and malunion in displaced SCHF (Vallila et al. 2015).

The risk of Volkmann’s ischemic contracture in displaced SCHF has fallen to nearly zero with internal fixation and adequate assessment of peripheral circulation and postoperative pain (Bashyal et al. 2009, Scannell et al. 2013). Displaced SCHFs carry the highest risk of nerve injury (up to 11%) among all pediatric fractures (Omid et al. 2008, Bashyal et al. 2009, Babal et al. 2010). Most nerve injuries are caused by the fracture itself, but up to a 4% incidence of iatrogenic injuries related to pin fixation have been reported (Gosens and Bongers 2003, Bashyal et al. 2009, Babal et al. 2010). The majority of nerve injuries associated with SCHF appear to resolve spontaneously within a few months (Dormans et al. 1995, Gosens and Bongers 2003, Ramachandran et al. 2006, Guner et al. 2013).

Quality of reduction has traditionally been assessed by radiographs. Frontal alignment can be evaluated by measuring the Baumann angle (BA), which is the angle between the long axis of the humeral shaft and the growth plate of the lateral humeral condyle with reported normal values between 64° and 81° (Williamson et al. 1992, Dai 1999, Shank et al. 2011, Flynn et al. 2015). The most common way to register sagittal alignment is to record whether the anterior humeral line (AHL) passes through the anterior or middle third of the ossification center of the capitulum (Herman et al. 2009, Flynn et al. 2015). Reliability of these radiographic indexes has been questioned (Silva et al. 2010), and it has been suggested that it is better to record the quality of reduction peroperatively by clinical comparison with the healthy side (Simanovsky et al. 2007, Tuomilehto et al. 2016).

The remodeling capacity of the distal humerus is limited (Otsuka and Kasser 1997, Omid et al. 2008, Flynn et al. 2015). Malunion in the frontal plane has been considered predominantly as a cosmetic disability, although elbow pain and dysfunction as well as an increased risk of lateral humeral condyle fractures have been reported (Guven et al. 2015). Sagittal plane malunion can lead to permanent changes in elbow range of motion (ROM) (Sinikumpu et al. 2016). Long-term outcome of SCHF is usually assessed clinically by Flynn’s criteria (Flynn et al. 2015), which define unsatisfactory results as more than 15˚ asymmetry in elbow ROM or carrying angle (CA). The subjective outcome has been evaluated with validated scoring systems such as the Pediatric Outcome Data Collection Instrument (PODCI), the Mayo Elbow Performance score, and QuickDASH. The limitation of these validated outcome measures is the absence of questions concerning cosmetic outcome, which in our opinion is a weakness, since the most common complication of SCHF, cubitus varus, is mainly considered a cosmetic problem.

Long-term outcome in displaced SCHF has not been well documented, however. To our knowledge, this is the largest follow-up study of operatively treated SCHF in children. Second, our goal was to study whether primary treatment outcome parameters have a prognostic value of long-term outcome of SCHF.

## Patients and methods

**Figure 1.**
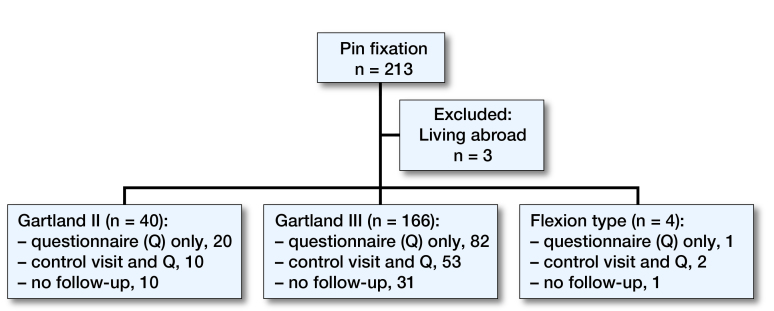
Operatively treated supracondylar humerus fractures 2002–2006. Patients’ participation in the study and fracture classification by Gartland.

During the 5-year study period in 2002–2006, 210 domestic SCHF (Gartland III 79%, Gartland II 19%, and flexion type 2%) were pin fixed in the Children’s Hospital, Helsinki (Figure 1). 115 (55%) patients were boys and 133 (63%) fractures left-sided. Mean age at the time of fracture was 7.2 (1.8–14.1) years. Trauma mechanisms were falling from height in 53%, sporting activities in 30% and falling on the level in 15%. 10 (5%) of the fractures were open fractures. The main surgeon was a consultant in 60 (29%) cases (8 consultants, mean number of operations per surgeon 8 (2–19)) and a registrar in 150 (71%) cases (31 registrars, mean number of operations per surgeon 5 (1–20)). Registrars performed 59 (28%) operations alone without consultant supervision.

14 (7%) patients had open reduction (anterior 11, lateral 2, medial and lateral approach 1). Osteosynthesis was performed through a small skin incision percutaneously with crossed pins in 200 patients and with laterals pins in 10 patients. The brachial artery of 1 patient was repaired. Volkmann’s contracture did not develop in any of our patients. 2 patients were primary operated by a registrar alone and reoperated within a week because of unsatisfactory primary reduction. 3 patients had their primary operation elsewhere and were reoperated in our institution within a week because of unsatisfactory primary reduction. 1 patient had a deep infection and an additional 7 patients (together 4%) had a superficial pin-track infection. Corrective osteotomy was performed in 2 patients (primarily operated by a registrar) due to gun-stock deformity after 3 and 8 years from the fracture.

36 (22%) of the 166 patients with Gartland III fractures had clinical findings of either median (19), ulnar (7), radial (7), or median and ulnar (3) nerve injury at discharge (even minor findings were recorded). Normal motor and sensory findings were recorded in 13 of these 36 patients preoperatively (1 median and ulnar, 3 ulnar, 3 radial, and 6 median nerve palsies). Electromyography (EMG) was undertaken on 18 of these 36 patients with no (11) or partial recovery within 3–6 months. 1 patient’s (primary treated by a consultant) median nerve was found partially entrapped in the fracture gap, released and repaired with subtotal recovery 1 year after fracture. All other patients’ sensorimotor functions recovered. Permanent nerve injury rate as a complication of treatment was thus 0.4%.

Postoperative radiographs of 197 (94%) patients were analyzed by a pediatric radiologist and an orthopedic registrar (NT). At fracture union alignment was regarded as satisfactory (BA within ±10˚ of reported normal range and AHL crossed capitulum) in 150/184 (82%) of the patients’ radiographs (13 patients did have either failed sagittal or coronal view radiographs). Grade II fractures healed in better alignment than grade III fractures (satisfactory result in 27/28 vs. 121/152, p = 0.03). The intra- (r = 0.91 and 0.91) and inter-observer (r = 0.92) reliability of Baumann angle measurement was considered excellent (Pearson’s correlation coefficient).

**Figure 2.**
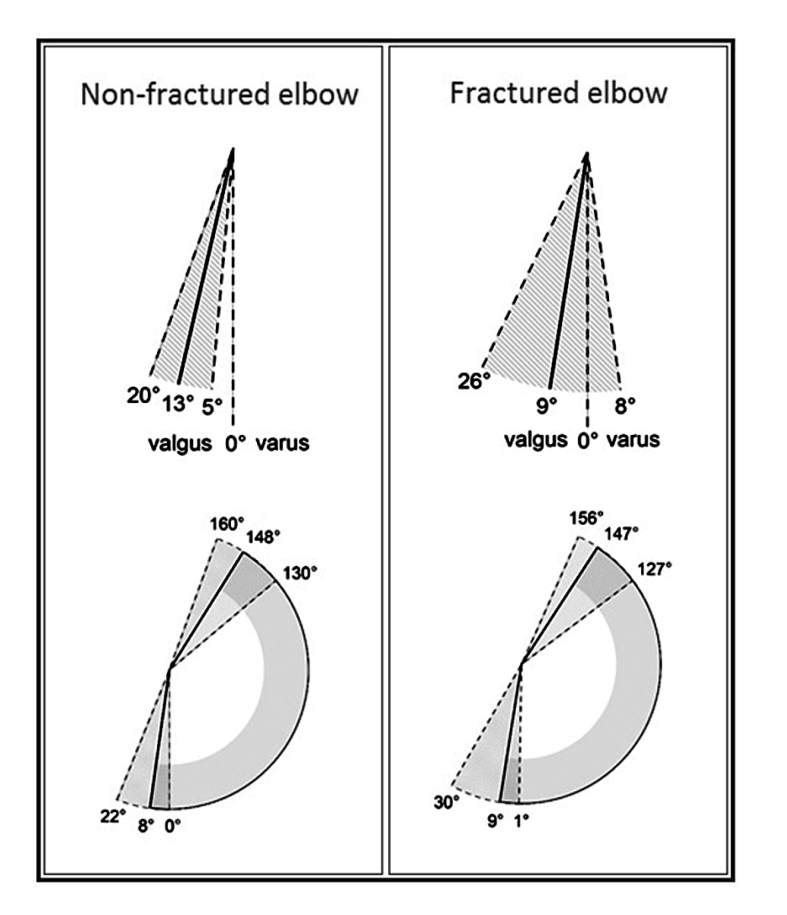
Carrying angle and elbow range of motion in 65 patients at mean 9 years after pin fixation of supracondylar humerus fracture.

After a mean follow-up time of 9 (7–12) years, 168 (80%) patients answered a questionnaire regarding appearance, function, and pain (scale 0–10) of the fractured elbow and symmetry of ROM and CA of their elbows (Table 1). 29 of the 36 nerve injury patents answered the questionnaire. 65 (31%) patients also attended for clinical examination performed by NT (Figure 1). ROM and CA of both elbows were measured with a goniometer (Figure 2). Radiographs of both elbows were taken if asymmetry in ROM or CA exceeded 10˚. Lengths of both arms were measured from the tip of acromion to the distal end of the lateral humeral condyle, and forearms from the tip of the olecranon to the distal end of the ulna, respectively.

**Table 1.** Questionnaire and answers (patients n = 168)

				Answers	
	Questions		Answer options	“yes”	mean (range)
1.	How satisfied are you with the appearance of the fractured elbow?	0–10	Very unsatisfied—very satisfied		8.7 (2–10)
2.	How satisfied are you with the functional outcome of the fractured elbow?	0–10	Very unsatisfied—very satisfied		9.0 (2–10)
3.	Do you have any pain at rest in the fractured elbow?	no/yes		7	
	If you answered yes, how intense has the pain been in the past week?	0–10	No pain—worst pain you can imagine		3.0 (1–8)
4.	Do you have any pain in motion in the fractured elbow?	no/yes		21	
	If you answered yes, how intense has the pain been in the past week?	0–10	No pain—worst pain you can imagine		3.9 (1–8)
5.	Are the carrying angles of your elbows symmetrical?	no/yes		140	
6.	Do your elbows flex symmetrically?	no/yes		149	
7.	Do your elbows extend symmetrically?	no/yes		132	

Of the 42 patients who did not participate in this study, 12 had postoperative follow-up elsewhere, 16 had no further controls after pin removal, 4 were followed up to 6–12 weeks to ensure fracture union and recovery of elbow motion and 1 up to 5 years because of valgus deformity. The remaining 9 patients who did not answer the questionnaire were seen regularly until satisfactory recovery of their nerve palsy (mean follow-up 6 months (1 month–2 years)).

There was no difference in radiological outcome at union between patients who answered only the questionnaire and those that also attended the clinical follow-up examination or between patients who participated in the study and those who did not.

Statistics analysis was performed using Fisher’s exact and Wilcoxon’s test. P-values ≤0.05 are considered statistically significant.

### Ethics, funding, and potential conflicts of interest

This study was approved by the local ethics committee (number 346/13/03/03/2012). Päivikki and Sakari Sohlberg Foundation, Finska Läkaresällskapet and Vappu Uuspää Foundation supported this study. No competing interests declared.

## Results

Mean subjective score for appearance was 8.7 (2–10) and for function 9.0 (2–10) according to the 168 answered questionnaires (Table 1). Fracture type, patient’s sex, and AHL in relation to capitulum did not affect the results. Mean functional scores were lower either in the 31 patients who were older than 10 years at the time of fracture (8.4, p = 0.01) and/or in the 29/36 patients who had nerve injuries (8.6, p = 0.05). Mean subjective scores for appearance were lower in the 14 patients who had had open reduction (7.8, p = 0.03) and in 9 patients who had BA values exceeding normal values by 10˚ (7.1, p = 0.02) (Figure 3, see Supplementary data). Open reduction patients’ AHL crossed the capitulum in 11/12 and BA was within 10˚ of the normal range in 11/12 cases. One or both subjective scores of 13 (8%) patients were below 6. 8 of these patients attended a control visit, and 5 of 8 had asymmetry of elbow ROM or CA or both of more than 10˚ (p = 0.002). 11 of these 13 patients were operated by a registrar (alone 5, under supervision 6) (p = 0.4).

Elbow pain either at rest (PIR) or in motion (PIM) or both was reported by 14% of the 168 patients who answered the questionnaire (PIM by 13% (median score 4 (1–8)) and PIR by 4% (median score 2.5 (1–8)) (Table 1). Pain was not related to fracture type or sex, but was more common in patients with nerve injuries (8/29, p = 0.04), in patients older than 10 years of age at the time of fracture (7/31, p = 0.2), in patients with elbow flexion deficit (> 10˚; 2/4, p = 0.2), and in patients with elbow CA asymmetry exceeding 10˚ into varus (3/9, p = 0.4).

Asymmetric elbow ROM was reported by 28% (extension 21%, flexion 11%) and CA by 17% of the 168 patients who answered the questionnaire (Table 1). Fracture type or radiographic findings at the time of fracture union did not affect the results, but asymmetric elbow ROM was more frequently experienced by girls (girls 29/75 vs. boys 18/93, p = 0.01). Subjective assessment of elbow ROM and CA of the 65 patients who attended the clinical examination proved to be unreliable in minor (< 11˚), but half as often recognized in more severe (> 10˚) asymmetry (Table 2).

**Table 2.** Patients’ subjective estimation compared with the control visit findings (n = 65)

		Asymmetry	Unrecognized asymmetry >5°			Recognized asymmetry >5°		
		≤ 5˚	6–10°	11–15°	> 15°	6–10°	11–15°	> 15°
Flexion deficit		4	1	2	0	1	1	1
Extension	deficit	0	1	0	0	1	1	0
	hyper	9	11	0	0	4	1	0
Carrying angle	varus	2	8	2	1	4	4	2
	valgus	0	0	0	0	2	0	0

Long-term outcome of elbow ROM was good or excellent in 60/65 and CA in 56/65 of the follow-up patients using Flynn`s criteria (Table 3). ROM asymmetry exceeding 10˚ was registered in 5/65 of patients (4 elbow flexion deficit, 1 hyperextension) attending for clinical examination. AHL crossed capitulum in 4 of these 5 patients’ radiographs at fracture union (1 patient’s lateral view radiograph was unusable). Elbow flexion deficit exceeding 10˚ correlated with lower subjective functional outcome (mean score 5.5 (3–10) p = 0.03; Figure 4, see Supplementary data). 4 of these 5 patients were operated by a registrar (alone 1, under supervision 3, p = 1.0).

**Table 3.** Long-term outcome by Flynn’s criteria at follow-up visit (n = 65)

Fracture type	Loss of motion			Loss of carrying angle		
(Gartland)	II	III	Flexion	II	III	Flexion
	n = 10	n = 53	n = 2	n = 10	n = 53	n = 2
Satisfactory						
Excellent (0–5˚)	8	46	2	6	38	1
Good (6–10˚)	1	3	0	0	10	1
Fair (11–15˚)	0	4	0	3	3	0
Unsatisfactory						
Poor (> 15˚)	1	0	0	1	2	0

CA asymmetry to varus exceeding 10˚ was measured in 9/65 patients attending for clinical examination. None of these 9 patients had BA over 10˚ outside the normal range or radiological evidence of malrotation at fracture union. CA varus asymmetry exceeding 10˚ correlated with decreased subjective cosmetic (p = 0.005) and functional (p = 0.004) outcomes (Figures 5 and 6, see Supplementary data). All of these 9 patients were operated by registrars (alone 6, under supervision 3, p = 0.06).

No asymmetry (> 5˚) in supination or pronation was found. Lengths of arms and forearms were equal. Radiographs of both elbows were taken in 12/65 patients (10 due to varus deformity and 2 due to flexion restriction). Signs of avascular necrosis or degenerative changes were not found.

Complication rate (iatrogenic nerve injury, re-reduction, deformity corrected by osteotomy, CA or ROM asymmetry >10˚) was greater in patients operated by a registrar (17/150 vs. 2/60, p = 0.1).

## Discussion

We have assessed long-term outcome of pin-fixed SCHF in Helsinki University Children’s Hospital, which is the largest institution treating pediatric fractures in Finland and the only hospital in Helsinki treating displaced SCHF in children. The Children’s Hospital is also the largest teaching hospital of pediatric orthopedics in Finland and therefore the majority (71%) of the patients in these series were operated by registrars, which should be taken into account since the quality of fracture reduction and osteosynthesis obviously affects the outcome in treatment of SCHF. In fact, the risk of complication was higher and the long-term outcome was poorer in patients operated by registrars compared with consultants in our study, although the differences did not reach statistical significance.

Volkmann’s contracture and permanent iatrogenic nerve injuries can be regarded as complications of treatment and their prevalence should be zero (Vallila et al. 2015). We consider a deep infection rate of less than 1% acceptable. Flynn’s criteria are generally used in order to assess the quality of treatment regarding elbow ROM and CA (Flynn et al. 2015). During the study period in 2002–6, the rate of Volkmann’s contracture was zero, and the risk of deep infection and permanent treatment-related nerve complications was low (< 1%) at our institution.

Sinikumpu et al. (2016) have reported 12 years’ long-term subjective outcome of 81 patients assessed by Mayo elbow performance score, but most of the patients had non-displaced SCHFs that were treated non-operatively. The Mayo score in the 25 patients with grade III fractures was excellent (mean 93), but range was not reported. In their minimum of 18 months’ follow-up study of a total of 154 Garland grade III SCHF, Wang et al. (2017) evaluated the long-term outcome of 33 neurovascular injury patients compared with patients with intact neurovascular findings. They used the Pediatric Outcomes Data Collection Instrument (PODCI) and Quick Disabilities of the Arm, Shoulder, and Hand (QuickDASH) outcome measures, both indicating excellent function. They found no statistically significant differences in outcome measures between the neurovascular injury patients and those with intact findings. No differences in outcomes were identified based on age, fracture site, sex, weight, direction of displacement, or operative technique in neurovascular injury patients. The authors speculated that a difference in the PODCI and DASH scores among groups could have been missed.

In our study, considering subjective scores 0–5 unsatisfactory and 6–10 satisfactory, 92% of our patients who answered the questionnaire had satisfactory results. Subjective cosmetic outcome correlated negatively with open reduction, which is most likely due to the scar. The number of patients with unsatisfactory subjective results could have probably been smaller with better quality of fracture reduction and pin fixation (satisfactory alignment at fracture union in 82% of patients in our study). Radiographic measurements had little value predicting subjective or objective outcome. This is most likely due to relatively large normal variation (almost 20°) and intra- and inter-rater errors (up to 7°) of measuring BA (Silva et al. 2010), although good inter- and intra-rated reliability in our study, as well as difficulties in obtaining true lateral radiographs of SCHF (Simanovsky et al. 2007, Guven et al. 2015) rendered radiographic assessment of fracture alignment unreliable. Previous follow-up studies have not analyzed their quality of reduction at fracture union by comparing their radiological measurements with reported normal values (Guven et al. 2015, Sinikumpu et al. 2016).

Sinikumpu et al. (2016) reported, also from Finland, that 9/25 of patients experienced pain at least 10 years after grade III fractures, whereas after grade I or II fractures the pain prevalence did not differ from normal age-matched controls. Fewer patients (14%) reported pain in our study without correlation to fracture grade but in association with nerve injury.

Guven et al. (2015) reported good or excellent functional and cosmetic long-term outcome (mean 22 years of follow-up) using Flynn’s criteria in 33 and 43 of their 49 SCHF patients with open reduction and cross-pin fixation (low clinical follow-up rate of 12%). In the study by Sinikumpu et al. (2016) (follow-up rate 76%), good or excellent results were achieved in 22 of 33 patients with grade II and III SCHF treated by either percutaneous or open pin fixation after minimum follow-up of 10 years. Our results (clinical follow-up rate 31%) were clearly better with good or excellent cosmetic and functional outcome in 56 and 60 of the 65 patients who were clinically examined at mean follow-up of 9 years. It has been recently reported by Vallila et al. (2015) that quality of treatment in distal humerus fractures correlates positively with the experience of the treating institution. Our results support this finding, since the annual number of pin-fixed SCHFs in our institution is 3-fold compared with the number reported in the study of Guven et al. (2015) from Istanbul and 10-fold compared with the earlier report from the joint study of two other institutions in Finland (Sinikumpu et al. 2016).

In Simanovsky’s study (2007) of 17 patients with malunited SCHF, 3 of 10 patients with elbow flexion restriction exceeding 5˚ had not recognized the ROM asymmetry and in 7 patients mild (up to 7˚) hyperextension of the elbow was registered. Only restricted elbow flexion was considered as a functional problem. In our study, only one-third of patients with minor elbow ROM deficiencies (less than 10˚ asymmetry) had noticed the asymmetry, whereas two-thirds of patients with more pronounced asymmetry were aware of CA or ROM discrepancy between their elbows (see Table 2). BA of more than 10˚ outside the normal reported range measured at fracture union was predictive of gun-stock deformity. Our results are in accordance with Simanovsky’s findings that decreased elbow flexion is a functional problem. We suggest that in the future only good and excellent results according to Flynn’s criteria should be classified as satisfactory, since asymmetry exceeding 10˚ in elbow ROM and CA was clearly associated with poorer subjective results in our study.

Limitations of our study are its retrospective nature and that only 31% of the patients attended for clinical follow-up examination. On the other hand, 80% of the patients answered the questionnaire and there was no difference between fracture types or quality of primary treatment between patients who participated in either part of the study with those who did not.

In summary, long-term cosmetic and functional outcome in SCHF are satisfactory with few exceptions if elbow ROM and CA can be restored within 10˚ of the uninjured elbow. Patients’ subjective estimation of minor (< 10˚) asymmetry in elbow ROM and CA is unreliable. Nerve injuries can cause long-term pain.

### Supplementary data

Figures 3–6 are available in the online version of this article, http://dx.doi.org/10.1080/17453674.2018.1438765.

A pediatric radiologist, Reetta Kivisaari, analyzed postoperative and control-visit radiographs. 

NT participated in planning the study, collected patient data, examined patients at the control visit and analyzed all radiographs and stored fluoroscopic images with the pediatric radiologist. AS participated in planning the study and editing the manuscript. AY participated in planning the study and is the main writer of the manuscript together with NT.

*Acta* thanks Vera Halvorsen and Klaus Parsch for help with peer review of this study.

## Supplementary Material

IORT_A_1438765_SUPP.PDF
