# Supplementary material for: 9 years’ follow-up of 168 pin-fixed supracondylar humerus fractures in children
Source: Acta Orthop. 2018 Feb 16;89(3):351–6. doi: 10.1080/17453674.2018.1438765 (PMC6055784; doi:10.1080/17453674.2018.1438765)
Supplement: IORT_A_1438765_SUPP.PDF [file IORT_A_1438765_SM9563.pdf]

Supplementary data

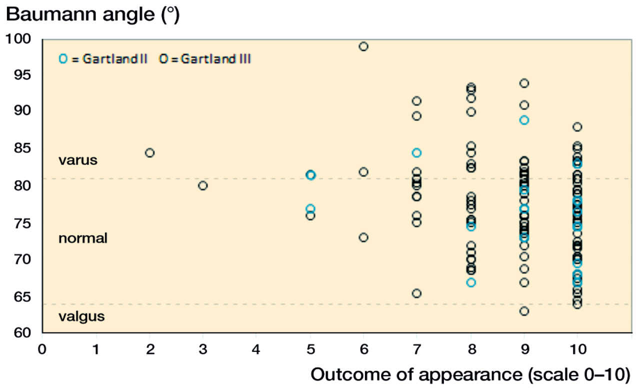

Figure 3. Subjective cosmetic result in correlation with Baumann angle (BA) at fracture union (n = 141).

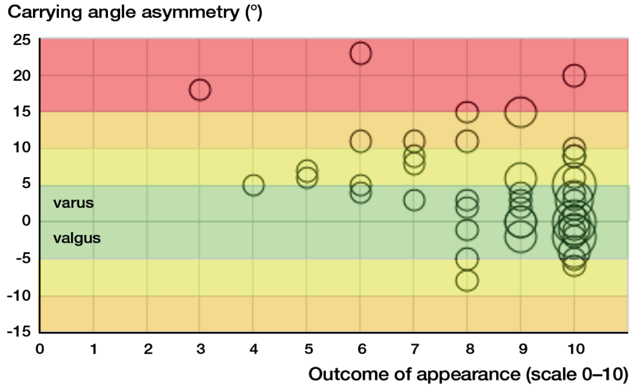

Figure 5. Subjective cosmetic result in correlation with carrying angle (CA) asymmetry (n = 65, dots represent patients: small dot = one patient, larger dots = several patients).

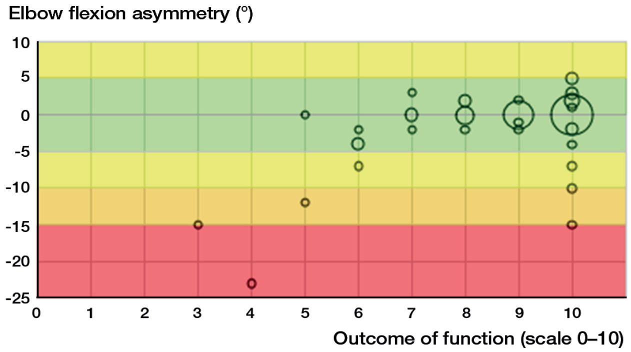

Figure 4. Subjective functional result in correlation with elbow flexion deficit (n = 65, dots represent patients: small dot = one patient, larger dots = several patients).

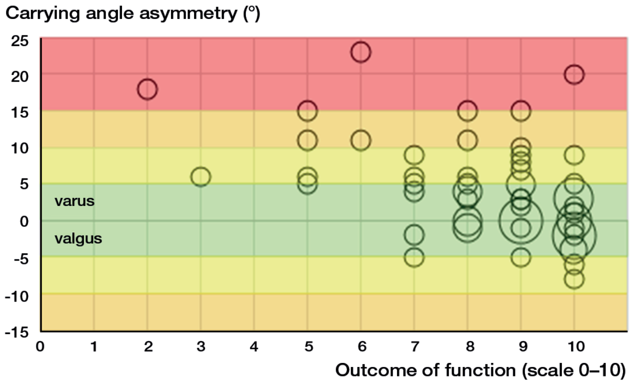

Figure 6. Subjective functional result in correlation with carrying angle (CA) asymmetry (n = 65, dots represent patients; small dot = one patient, larger dots = several patients).
